# Interactive Effects of Precipitation and Nitrogen on Soil Microbial Communities in a Desert Ecosystem

**DOI:** 10.3390/microorganisms13061393

**Published:** 2025-06-14

**Authors:** Qianqian Dong, Zhanquan Ji, Hui Wang, Wan Duan, Wenli Cao, Wenshuo Li, Yangyang Jia

**Affiliations:** 1 College of Ecology and Environment, Xinjiang University, Urumqi 830046, China; dongqianqian@stu.xju.edu.cn (Q.D.); Jizhanquan@stu.xju.edu.cn (Z.J.); wanghui@stu.xju.edu.cn (H.W.); 107552401736@stu.xju.edu.cn (W.D.); caowenli@stu.xju.edu.cn (W.C.); 19606365367@163.com (W.L.); 2 Key Laboratory of Oasis Ecology, Xinjiang University, Urumqi 830046, China

**Keywords:** increased precipitation, nitrogen deposition, soil microbial community, phospholipid fatty acid, Gurbantunggut desert

## Abstract

Increased precipitation and nitrogen (N) deposition critically influence ecological processes and stability in desert ecosystems. Studying how the soil microbial community responds to these climatic changes will improve our understanding of the impacts of climate changes on arid environments. Therefore, we conducted a field experiment in the Gurbantunggut Desert, applying phospholipid fatty acid (PLFA) analysis to assess the responses of soil microbial community to climate change. We found that in years with normal precipitation, increased precipitation promoted soil bacterial growth, whereas in drought years, increased N deposition promoted soil bacterial growth more effectively. Although soil microbial diversity did not change significantly overall, it decreased with increasing N deposition. Random forest analysis and linear regression analysis indicated that soil pH and microbial biomass carbon (MBC) were the main drivers for the changes in soil microbial community. Structural equation modeling (SEM) further revealed that increased precipitation increased soil Gram-positive bacteria (G^+^) by raising soil MBC, while decreasing soil Actinomycetes (Act), fungi, and Dark Septate Endophyte (DSE). In contrast, increased N deposition affected soil microbial community by altering soil pH and MBC. Our results highlight the synergistic effects of increased precipitation and N deposition on soil microbial community structure. Further research should pay more attention to the effects of climate changes on soil microbial communities with long-term monitoring to confirm our findings across different ecosystems.

## 1. Introduction

Desert ecosystems cover about 17.64 × 10^6^ square kilometers of land (12%) and support the survival and development of 2 billion people globally [1]. This environment is extremely fragile, characterized by high temperatures, drought, and sparse vegetation under intense evapotranspiration [2]. In recent years, increased precipitation and N deposition have emerged as key issues of climate change affecting desert ecosystem functioning [3]. Soil microorganisms play a vital role in maintaining diverse ecosystem functions and are actively involved in multiple ecological processes such as organic matter decomposition, nutrient cycling, and energy flow [4]. While soil microbial community composition and structure are strongly influenced by water availability and nutrient supply, their interactions determine the number of soil microorganisms and their community structure, especially in desert soils [5].

Changes in precipitation are among the major factors influencing the functions and activities of soil microorganisms, and the responses of the soil microbial community are highly modulated by the degree of precipitation variability [6]. It is indicated that increased precipitation promoted the relative amount of soil fungi compared to soil bacteria, and posed limited effects on soil Gram-positive bacteria (G^+^) due to their good osmoregulatory capacity [7]. In addition, changes in precipitation regulate soil microbial activity through a dual pathway: on the one hand, it directly impacts the soil microbial metabolism by affecting soil pH and nutrient availability (e.g., N and phosphorus mineralization) [8]; on the other hand, water augmentation improves soil aeration and water content, accelerates nutrient release (e.g., soluble N accumulation), and indirectly stimulates soil microbial growth [9]. In desert grassland ecosystems, increased precipitation significantly increases the species richness as well as the evenness of bacteria in the soil [5,10]. Similarly, in desert ecosystems, elevated soil pH levels due to increased precipitation promote the activity of soil microorganisms, which are more tolerant of alkali (e.g., actinomycetes) and inhibit the activity of soil fungi in acidic environments [11]. The processes by which precipitation changes affect soil microbial communities and their underlying mechanisms are complex, especially in nutrient-limited desert areas. Therefore, this study could provide theoretical guidance by exploring the underlying influence mechanisms of climate change on soil microbial communities in desert ecosystems.

Increased N deposition has been proven to be another key factor influencing soil microbial communities and has been extensively studied with positive, negative, and limited effects across different ecosystems [4,12]. More importantly, the effects of increased N deposition on soil microbial communities differ among different soil microbial species. Increased N deposition reduced the soil microorganisms’ biomass and decreased the ratio of soil fungal to bacterial biomass in grassland ecosystems, indicating that increased N deposition posed negative effects on the soil microbial community, which might be related to soil acidification and nutrient changes [13]. Increased N deposition significantly reduced the soil bacterial community species richness, but posed limited effects on soil fungal community species richness, indicating soil bacterial community responds more sensitively to increased N deposition than the soil fungal community [14]. Furthermore, simulated N deposition significantly altered the community structure of arbuscular mycorrhizal fungi (AMF), but had no significant effects on their diversity in temperate and subtropical forest ecosystems [15]. Notably, soil microbial communities respond sensitively to increased N deposition in desert ecosystems where N is the limiting factor for ecological processes. It has been shown that increased N deposition resulted in a short-term increase in the diversity of soil bacterial and fungal communities, and particularly triggered significant increases in the relative abundance of those microbial taxa that can utilize N (e.g., actinomycetes) in desert ecosystems [16]. Increased N deposition could promote soil fungi growth under arid conditions because they are better adapted to the harsh environment with limited soil moisture and N contents than soil bacteria [17,18]. It has also been indicated that simultaneous alterations in precipitation and N could have more significant effects on the soil microbial community than individual factors. For example, increased N deposition reduced the species richness and diversity of the soil bacterial community, while increased precipitation positively affected the soil fungal community in semi-arid grassland ecosystems [11]. This interaction may also indirectly affect the soil microbial community by altering the availability of soil nutrients such as N and phosphorus [5,19]. Although the effects of increased N deposition on soil microbial community structure have been widely explored, few studies have been conducted in desert ecosystems, especially under the dual conditions of increased precipitation and N deposition [20]. Therefore, it is of great significance to carry out research to uncover the underlying influence mechanisms of increased precipitation and N deposition on soil microbial communities in desert ecosystems.

Desert ecosystems are normally with sparse vegetation and large areas of bare soil [1]. In this sense, the desert ecosystems provide an excellent platform to explore the real responses of soil microbial communities to climate change without the interference of plant communities, and shed light on the potential mechanisms of soil microorganisms’ responses to climate change in other ecosystems [3]. Furthermore, soil microorganisms could be adapted to exist in the harsh environmental conditions that are suitable for their life in desert ecosystems [3,21]. Considering this, the responses of soil microorganisms to climate change might be insensitive and inconsistent with other ecosystems and need to be further revealed. Additionally, soil microorganisms research has made fundamental contributions to our understanding of the responses of the soil microbial community to climate change, with the rapid technological development of molecular biology (e.g., quantitative real-time PCR) over the last decade [5,22]. In contrast to the DNA-based method, the phospholipid fatty acid (PLFA) analysis can provide more accurate responses of soil microbial communities to environmental perturbations due to the rapid degradation of PLFA after cell death and has been widely used [23]. It is the most reliable amount of variation in microbial abundance in terms of spatial and temporal trends.

Here, we conducted a four-year *in situ* experiment with increased precipitation and N deposition in Central Asia. The PLFA method was employed to reveal the changes in soil microbial community structure, and soil physicochemical parameters were tested to uncover the potential influence mechanism of increased precipitation and N deposition on soil microbial community structure in desert ecosystems. Our main aim was to address the following two questions: (1) How do increased precipitation, increased N deposition, and their interactions affect the soil microbial community through soil physicochemical properties? (2) Whether different soil microbial taxa respond differently to increased precipitation and increased N deposition?

## 2. Materials and Methods

### 2.1. Overview of the Study Area

This study was carried out in the Gurbantunggut Desert (44°26′ N, 87°54′ E), in northwestern China, Central Asia (Figure S1). This region belongs to a typical temperate desert ecosystem with an average elevation of 454 m. The mean annual temperature was 7.1 °C, and the mean annual precipitation was 215.6 mm over the past 40 years. Notably, the rainfalls of our sampling years (i.e., 2016 and 2017) were largely different. The rainfall for 2016 and 2017 was 203.7 mm and 126.8 mm, respectively, but the mean annual temperature for 2016 and 2017 was similar, at 6.3 °C and 6.1 °C, respectively (Figure S2). Thus, we defined 2016 as the normal precipitation year, but 2017 was a drought year (Figure S2). For the experimental design, W0 (natural precipitation) and W1 (30% increase) were treatment groups set within the same year rather than compared across years. Combined with the soil classification system of Bronick and Lal (2005), the soil type in this study area is gray desert soil. Its mechanical composition is dominated by sandy grains (>75%) with <8% clayey grains [24], and the content of soil organic matter is 2.21 ± 0.71 g/kg, with a soil pH of 8.8. Common widespread species include *Erodium oxyrrhynchum*, *Schismus arabicus*, *Corispermum lehmannianum*, *Tetracme recurvata*, *Eremopyrum orientale*, and *Malcolmia scorpioides*. The mean plant densities of the two years were 124 and 101 plants/m^2^; the mean cover values were 38.8–43.1% and 18.6–24.1%; and the aboveground biomasses were 129.03–166.15 g/m^2^ and 17.12–29.08 g/m^2^, respectively. Therefore, the Gurbantunggut Desert is a natural laboratory for studying the effects of climate change on soil microbial community composition.

### 2.2. Experimental Design

Based on the analysis of annual precipitation from 1980 to 2010 in the study area, the average annual rainfall was found to be 200 mm, and precipitation is expected to increase by 30% in the next 30 years [25]. Therefore, we simulated precipitation with treatment settings of natural precipitation (W0) and a 30% increase (i.e., 60 mm) in natural precipitation (W1). N deposition in the desert region of Xinjiang is about 15 kg N ha^−1^yr^−1^ [26], based on model projections. N deposition in 2050 is projected to be approximately double the current level, with continued increases in N deposition expected through the end of the century [27]. Therefore, we set up three N levels: N0: natural deposition; N1: medium N deposition (30 kg N ha^−1^yr^−1^ increase on natural basis); N2: high N deposition (60 kg N ha^−1^yr^−1^ increase on natural basis). Thus, the increased precipitation and N deposition complete block experiment included six treatments (W0N0, W0N1, W0N2, W1N0, W1N1, W1N2) with four replicates per treatment. A total of 24 experimental plots with a plot size of 10 m × 10 m and a 5 m isolation zone were set between each plot in 2014. To simulate the effects of changes in seasonal precipitation patterns, water and N deposition were examined in the spring, summer, and fall, with four simulations in each season at l-week intervals, for a total of 12 simulations per year, each simulating 5 mm precipitation. NH_4_NO_3_ was chosen to simulate increased N deposition based on the two considerations: (1) NH_4_^+^-N and NO_3_^−^-N are the main inorganic forms of the natural N deposition in the study area; (2) providing the two inorganic N forms at the same time can more faithfully reflect the ecological process of N deposition in arid zones, e.g., nitrification of the soil. Thus, the use of NH_4_NO_3_ can accurately simulate the actual N deposition scenarios, comprehensively assessing the effects of N deposition on the soil microbial community, and avoiding the microbial metabolism bias that may be caused by a single N form (e.g., NH_4_^+^ or NO_3_^−^ alone) [28]. In addition, for better N uptake by plants and soil, NH_4_NO_3_ was dissolved in 50 L of water (equivalent to 0.5 mm of precipitation) and sprayed evenly into the plots using a 3WBD-20 motorized backpack sprayer (Zhejiang WOSH Gardening Technology Co., Ltd., Taizhou, China) equipped with a TR-8002 fan-shaped atomizing nozzle (operating pressure 0.3–0.4 MPa, flow rate coefficient 0.8, droplet volume median diameter VMD = 250 ± 15. The control group was sprayed with an equal amount of water.

### 2.3. Sample Collection

Soil samples were collected from the 0–20 cm soil layer on May 25 of each year when aboveground plant biomass reached its peak in 2016 and 2017, respectively. After removing dead leaves from the soil surface, three soil cores (3 cm in diameter and 20 cm in depth) were collected according to the randomization method and uniformly mixed to form one composite sample after sieving by 2 mm mesh, obtaining a total of 24 composite samples per year (6 treatments × 4 replicates), for a total of 48 samples in two years. Soil samples were then divided into three parts: one part was naturally dried for the determination of soil physicochemical properties, the second portion was immediately placed in sterilized polyethylene bags and stored in a refrigerator at 4 °C until the soil microbial carbon and N were determined within one month, and the third part was stored at −80 °C for PLFA analysis.

### 2.4. PLFA Determination and Soil Parameters Measurements

For PLFA determination, eight grams of lyophilized soil samples were weighed and extracted twice with citrate buffer solution (0.15 M, pH 4.0, Sigma-Aldrich B9434; Merck KGaA, Darmstadt, Germany)-chloroform-methanol mixture in the ratio of 0.8:1:2 with shaking. The fatty acids were then separated on an SPE silica column, and the phospholipid fatty acids were alkaline methanolized with an internal standard of methyl n-nineteen adipate (19:0, Sigma CRM47885; Merck KGaA, Darmstadt, Germany, final concentration 10 μM). Finally, PLFA were detected by gas chromatography (GC-FID, Agilent 7890B, Santa Clara, CA, USA), and fatty acids were quantified in nmol/g using a fatty acid standard and microbial identification system (MIDI, Inc., Newark, DE, USA) [29]. Microbial taxa PLFA assays were categorized into six groups based on the method described by Bossio [30] and Yang et al. [31]: Gram-positive bacteria (i12:0, i13:0, i14:0, a15:0, i15:0, i16:0, a16:0, a17:0, i17:0, i18:0), Gram-negative bacteria (i15:0 3OH, 16:1ω 7c, 16:1ω9c, 17:1ω7c, 17:1ω8c, i17:0 3OH, 18:1ω7c, cy17:0), Actinomycetes (10Me16:0, 10Me17:0, 10Me18:0), Fungi (18:1ω9c, 18:2ω6,9, 18:3ω6c(6,9,12)), Arbuscular Mycorrhizal Fungi (AMF) (16:1 ω5c), Dark Septate Endophyte (DSE) (16:00:00, 18:00:00).

Soil microorganisms’ diversity was determined by calculating the Shannon Diversity Index (*H*) [32], Simpson’s index of dominance (*D*) [33], and Pielou’s homogeneity index to represent (*J*) [34]:

The Shannon–Wiener diversity index (*H*) is given as follows:

(1) $$H=-\sum_{i=1}^{s}\left(P_{i}lnP_{i}\right)$$

Simpson’s index of dominance (*D*) is as follows:

(2) $$D=1-\sum_{i=1}^{S}P_{i}^{2}$$

Pielou Uniformity Index (*J*) is as follows:

(3) $$J=\frac{H}{lnS}$$

where *P_i_* is the proportion of *i* characteristic PLFA to the total number of characteristic fatty acids, and *S* is the total number of PLFA species in the community.

Soil pH was determined using deionized water, mixed at a soil–water ratio of 1:5, and measured using a pH meter (Seven Easy, Mettler-Toledo, Greifensee, Switzerland). Soil organic carbon was determined using the oxidation method with a potassium dichromate-sulfuric acid solution. Soil ammonium N (NH_4_^+^-N) and nitrate N (NO_3_^−^-N) were determined using a flow analyzer (AA3, SEAL, Analytical, Southampton, UK; legacy Bran+Luebbe Corp., Delavan, WI, USA). The soil microorganism carbon and nitrogen levels were determined by chloroform fumigation-K_2_SO_4_ leaching.

### 2.5. Statistics and Analysis of Data

First, two-way ANOVA was applied to evaluate the effects of increased precipitation, increased N deposition, and their interaction on the soil physicochemical factors and microbial community structure and diversity (Tables S1–S3). The significance of differences was tested using the least significant difference (LSD) method (*p* < 0.05) with SPSS 19.0 software. Secondly, non-metric multidimensional scaling (NMDS) analysis based on the Euclidean distance was used to reveal the effects of increased precipitation and N deposition on the soil microbial community structure during the 2-year period in R 4.3.3. The relationships between soil microorganism functional groups and soil physicochemical factors were calculated by using the “pheatmap” package. In addition, the contribution of soil physicochemical properties to soil microbial communities under increased precipitation and N deposition was described using a random forest (RF) model (number of trees = 750, unlimited maximum depth, number of variables automatically selected at splitting) using the “randomForest” software package (v4.7-1.1). Based on the results of the random forest model, the “lmtest” software package (v0.9-40) was used to further reveal the linear correlation between soil pH, MBC, and soil microbial community. Finally, structural equation modeling (SEM) was developed to estimate the potential pathways of changes in soil microbial community due to increased precipitation and N deposition through soil physicochemical properties. SEM analyses were performed using the R package “lavaan” version 4.3.3. Other statistical analyses were performed using SPSS 19.0 (SPSS Inc., Chicago, IL, USA), and concept maps were drawn in PowerPoint (Microsoft Office Home and Student 2019).

## 3. Results

### 3.1. Effects of Increased Precipitation and N Deposition on Soil Physicochemical Properties

The effects of increased precipitation and N deposition on soil physicochemical properties were approximately the same in 2016 and 2017 (Table 1 and Table S1). SOC, NO_3_^−^-N, and NH_4_^+^-N contents were significantly increased by increased N deposition, but increased precipitation posed limited effects in both years (Table 1 and Table S1). Soil pH was only significantly decreased as a result of increased N deposition in 2016 (Table 1 and Table S1). Notably, increased precipitation significantly decreased soil MBC and MBN contents in both years (Table 1 and Table S1). Furthermore, there were significant interactions of increased precipitation and N deposition on soil MBC and MBN contents, i.e., increased N deposition significantly increased soil MBC and MBN contents under the ambient precipitation treatment, while soil MBC and MBN contents reduced under increased precipitation (Table 1).

### 3.2. Effects of Increased Precipitation and N Deposition on Soil Microbial Community Diversity and Structure

**Figure 1:**
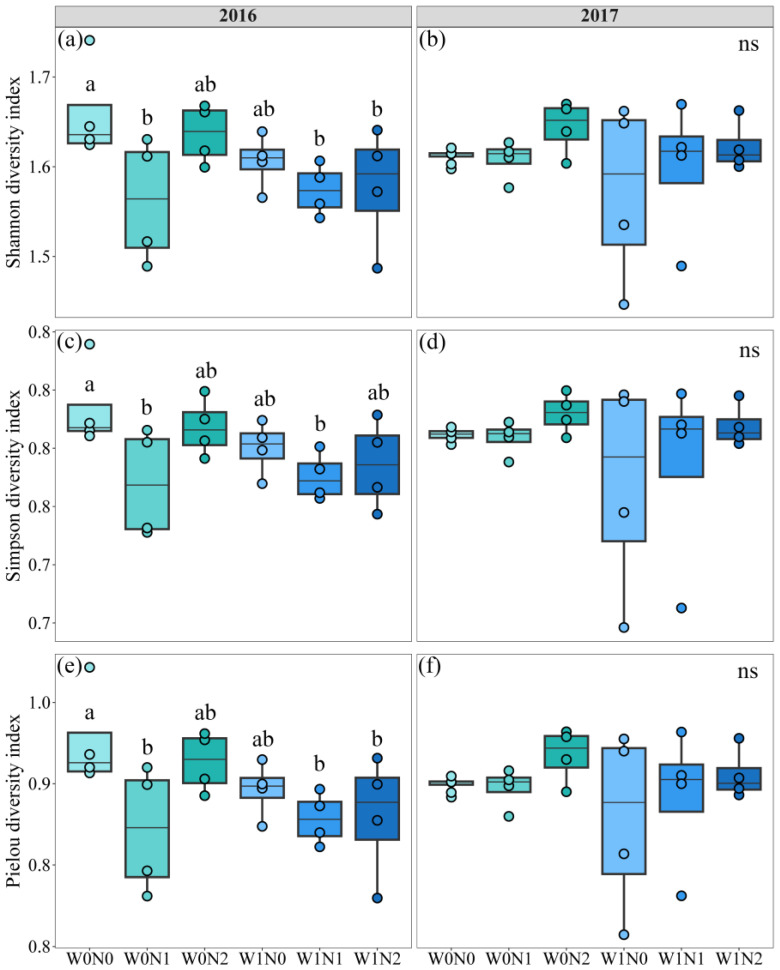
Effects of increased precipitation and N deposition on soil microbial community diversity in 2016 (**a**,**c**,**e**) and 2017 (**b**,**d**,**f**). All data are presented as the mean ± standard error. Note: Different lowercase letters in the figure indicate significant differences between treatments (*p* < 0.05). W0, Natural precipitation; W1, 30% increase in natural precipitation; N0, Natural deposition; N1, Medium N deposition; N2, High N deposition. ANOVA results are shown in Table S4.

**Figure 2:**
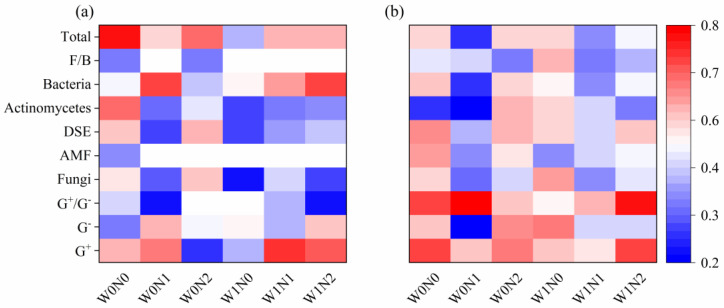
Results of changes in soil microbial content under increased precipitation and N deposition in 2016 (**a**) and 2017 (**b**). Note: G^+^/G^−^, Gram-positive bacteria/Gram-negative bacteria; AMF, Arbuscular Mycorrhizal Fungi; DSE, Dark Septate Endophyte; F/B, Fungi/Bacteria.

We identified notable changes in soil microbial diversity with increased N deposition in 2016, but the effects were less pronounced in 2017, as revealed by the results of two-way ANOVA (Table S4). Increased precipitation had limited effects on soil microbial community diversity in 2016 and 2017 (Figure 1; Table S4). Furthermore, the Shannon diversity index and Pielou diversity index decreased by 5.87%, 5.13%, and 4.90% under W0N1, W1N1, and W1N2 treatments, respectively, compared to W0N0, indicating that N deposition significantly reduced microbial diversity (Figure 1a,e; Table S5). Simpson’s diversity index was significantly reduced by 3.25% and 2.97% in W0N1 and W1N1 treatments, compared to W0N0, respectively, indicating that low N deposition significantly reduced microbial diversity. (Figure 1c; Table S5). Although the relative abundance of soil Act and DSE decreased with increasing N deposition, the effects of increased precipitation and N deposition on soil microbial community structure were not significant according to the NMDS analysis (Figures S3 and S4). In both years, increased precipitation significantly promoted soil G^−^ content (Figure 2; Table S2). In 2016, soil fungi and Act content decreased with increased precipitation, while soil bacteria content increased; in 2017, soil fungi and Act content increased significantly under the same precipitation treatments (Figure 2; Table S2). The effect of increased N deposition showed a concentration-dependent pattern: in both years, low N deposition decreased soil AMF, bacteria, and total PLFA contents in both years, while their contents increased under high N deposition; soil G^+^ contents increased under low N deposition in 2016, while their contents decreased under high N deposition (Figure 2; Table S2). In addition, increased N deposition significantly decreased soil F/B in both years, and the interaction effects of increased precipitation and N deposition significantly promoted soil G^+^/G^−^ (Figure 2).

### 3.3. Relationships Between Soil Microbial Community and Soil Physicochemical Properties

**Figure 3:**
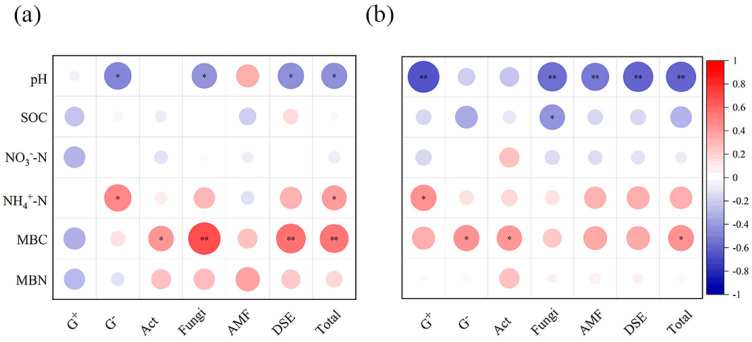
Correlation analysis of soil microbial PLFA content with soil physicochemical factors in 2016 (**a**) and 2017 (**b**), respectively. Note: SOC, Soil organic carbon; NO_3_^−^-N, Nitrate nitrogen; NH_4_^+^-N, Ammonium nitrogen; MBC, Microbial biomass carbon; MBN, Microbial biomass nitrogen; G^+^, Gram-positive bacteria; G^−^, Gram-negative bacteria; Act, Actinomycetes; AMF, Arbuscular Mycorrhizal Fungi; DSE, Dark Septate Endophyte. *, *p* < 0.05; **, *p* < 0.01.

Soil microorganism contents were significantly related to the soil physicochemical properties (Figure 3). In 2016, the contents of soil G^−^, fungi, and DSE were negatively correlated with soil pH; the contents of soil G^−^ were positively correlated with NH_4_^+^-N; and soil Act, fungi, and DSE were positively correlated with MBC (Figure 3a). In 2017, the contents of soil G^+^, fungi, AMF, and DSE were negatively correlated with soil pH; the contents of soil fungi were negatively correlated with SOC; and the contents of soil G^−^ and Act were positively correlated with MBC (Figure 3b). The contents of the total soil microbial community were negatively related to soil pH but positively related to soil MBC in 2016 and 2017 (Figure 3). Overall, soil microorganisms PLFA content showed strong correlations with soil pH and MBC.

**Figure 4:**
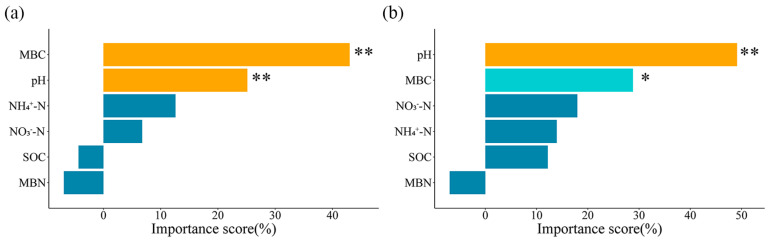
Contribution of soil physicochemical properties to soil microbial community in 2016 (**a**) and 2017 (**b**) based on random forest analysis. *, *p* < 0.05; **, *p* < 0.01.

The results of the random forest analysis further confirmed that soil MBC and pH were the main factors affecting the soil microbial community in both 2016 and 2017 (Figure 4). Notably, in order to further elucidate the influence mechanism, linear regression was employed to reveal the relationships between soil MBC, pH, and major microbial taxa (Figures S5 and S6). Linear regression results showed that the contents of soil G^−^, fungi, and DSE showed negative linear correlations with soil pH in 2016 (Figure S5b,d,f). Whereas in 2017, the contents of soil G^+^, fungi, AMF, and DSE were linearly and negatively correlated with soil pH (Figure S5a,d–f). Furthermore, we found that the contents of soil Act, fungi, and DSE showed positive linear correlations with soil MBC in 2016 (Figure S6c,d,f), and the contents of soil G^−^ and Act showed positive linear correlations with soil MBC in 2017 (Figure S6b,c).

### 3.4. Direct and Indirect Effects of Soil Microbial Community Drivers

**Figure 5:**
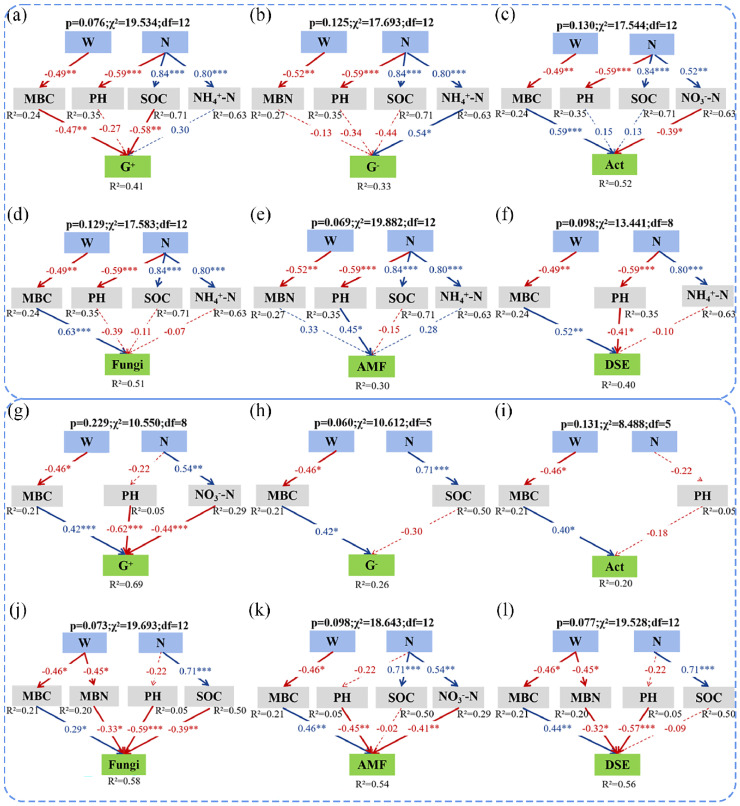
Structural equation modeling (SEM) illustrates the causal pathways through which increased precipitation and N deposition affected the soil microbial community in 2016 (**a**–**f**) and 2017 (**g**–**l**), respectively. Solid red and blue arrows indicate negative and positive significant effects, respectively, and dashed lines indicate non-significant effects. Numbers above the arrows indicate the magnitude of the standardized SEM coefficients (*, *p* < 0.05; **, *p* < 0.01; ***, *p* < 0.001). R^2^ values indicate the proportion of variance explained by each variable.

The best-fitting SEM further revealed the potential influence mechanism of increased precipitation and N deposition on soil microbial community structure (Figure 5; Figure S7). In 2016, increased precipitation increased soil G^+^ content by decreasing soil MBC, which was positively related to soil G^+^ (Figure 5a). While increased precipitation significantly decreased soil Act, fungi, and DSE content by reducing soil MBC, which was negatively related to soil Act, fungi, and DSE content (Figure 5c,d,f). By contrast, increased N deposition reduced soil AMF and DSE content by decreasing soil pH, which was negatively correlated with AMF and DSE content (Figure 5e,f). Increased N deposition significantly increased SOC, while the negative correlations between SOC and G^+^ resulted in the decline of soil G^+^ content with the increased N deposition (Figure 5a). And, increased N deposition reduces G^−^ and soil Act by increasing NH_4_^+^-N and NO_3_^−^-N, respectively (Figure 5b,c). In 2017, increased precipitation reduced soil G^+^, G^−^, Act, fungi, AMF, and DSE content by decreasing soil MBC, which was positively correlated with the content of each soil microorganism group (Figure 5g–l); However, increased precipitation increased soil fungi and DSE by decreasing MBN (Figure 5j,l). By contrast, increased N deposition significantly increased NO_3_^−^-N, but its negative correlation with soil G^+^ and AMF led to a significant decrease in both microbial taxa by increased N deposition (Figure 5g,k). And, increased N deposition significantly increased soil SOC content, while the negative correlation between SOC and fungi resulted in a decrease in soil fungi content with increased N deposition (Figure 5j). Structural equation modeling corroborated the random forest analysis, indicating that soil pH and MBC were the main drivers of the changes in soil microbial community structure in desert ecosystems (Figure S7).

## 4. Discussion

Changes in precipitation and atmospheric N deposition are important features of global climate change and key drivers regulating soil microbial community structure in desert ecosystems [3]. Soil microbial communities play integral roles in maintaining ecosystem functions and services, and desert ecosystems provide an ideal research area to explore the real responses of soil microbial communities to climate change without the buffering effects of plant communities [11,35]. In this study, we investigated the potential influence mechanisms of the effects of increased precipitation and N deposition on soil microbial communities based on the PLFA method in desert ecosystems. Increased precipitation and N deposition posed significant effects on the soil microbial community structure in the normal precipitation conditions but showed limited effects in drought years. Furthermore, soil pH and MBC content were the main drivers of the changes in the soil microbial community structure, i.e., increased precipitation altered soil microbial community structure by reducing soil MBC content, but increased N deposition altered soil microbial community structure by N-induced acidification.

### 4.1. Effects of Increased Precipitation on Soil Microbial Community

Changes in precipitation pose significant effects on altering soil microbial communities by changing the relative abundance of bacteria and fungi in favor of a particular microorganism’s taxon [8,36]. Contrary to our findings, in semi-arid grasslands where increased precipitation favored soil fungal dominance [37]. Our studies in the Gurbantunggut Desert showed a significant increase in G^−^ content due to their dependence on soluble carbon from enhanced SOC mineralization under wetter conditions [8,38]. In addition, the present study found that increased precipitation in normal-precipitation year decreased the relative abundance of soil fungi and Act, but increased the relative abundance of soil bacteria, which is consistent with the findings of Mao and Huang et al. [39,40]. The increase in precipitation altered the soil environmental conditions for soil microorganisms, especially soil fungi can transport water through mycelium insertion into the microporous water film, while soil bacteria depend on the movement of water to diffuse the substrate [41]. However, in drought years, increased precipitation raised the relative abundance of soil fungi and Act, which is consistent with the previous studies, which indicated that soil fungi showed greater tolerance to arid environments [42]. The possible reason may be that soil fungi can transport water and nutrients from resource-rich areas to resource-limited areas through the expansion of mycelial networks, while increased precipitation is more conducive to enhanced soil microbial activity and rapid substrate utilization [43,44]. Furthermore, our study further found that increased precipitation in both years decreased soil MBC contents, and subsequently reduced soil DSE and total PLFA, which is inconsistent with previous studies [45]. One previous study indicated that increased precipitation led to insufficient energy supply for soil microorganisms growth by reducing soil carbon source (i.e., MBC), and consequently leading to reductions in DSE and total PLFA content in soils [46]. Additionally, previous studies found that declines in soil MBC inhibit the growth of soil microorganisms through low adsorption of SOC and other low-molecular-weight organic matter in soils [47]. Recent studies agreed with our findings that increased precipitation accelerated soil material cycling and cation leaching, inhibited soil microorganisms activities by lowing soil pH, and subsequently reducing soil organic carbon pools and decreasing soil PLFA contents [41,48]. This study suggests that increased precipitation may activate synergistic strategies of microbial functions that have long been adapted to arid environments, e.g., increased bacterial and fungal content in the presence of increased precipitation, suggesting that moisture elevation may enhance mycorrhizal collaboration by promoting carbon substrate sharing. In addition, salinity is an important abiotic factor affecting soil microbes, and we should take these factors into account in the future [28].

Additionally, increased precipitation is also one of the main factors affecting soil microbial community diversity. In this study, we found that soil microbial community diversity presented a decreasing trend under increased precipitation conditions in 2016, which is in line with previous studies [11,49]. The study showed that increased precipitation in the early part of high-precipitation years exacerbated soil acidification, which in turn led to a downward trend in soil microbial community diversity [50]. However, it is worth noting that the effects of increased precipitation on soil microbial community diversity were not significant in 2017. This is possibly because soil microorganisms in desert ecosystems may have adapted to harsh environments and with high resistance to environmental perturbations [12,51]. But, with the continued progress of environmental perturbations over time, increased precipitation could pose significant effects on soil microbial community diversity [13,52], although sparse vegetation highly reduces the interferences of plant and rhizosphere processes on soil microbial community, the extremely limited soil water could be preferentially absorbed by the pioneer plants, and thus reducing the impacts of precipitation changes on soil microbial community [53,54].

### 4.2. Effects of Increased N Deposition on Soil Microbial Community

N is one of the key nutrients required by soil microorganisms and a primary factor limiting the activity and structure of the soil microbial community in desert ecosystems [3,12]. Increased N deposition provides additional nutrients that could surely promote soil microorganisms’ activities and numbers [55]. Our results showed that soil AMF, bacteria, and total PLFA contents were increased under high N treatment, which is in line with the results of the previous studies [8,56]. Ullah et al. found that under relatively low N conditions, soil microorganisms may not be able to obtain enough N to support their growth and reproduction due to the limited N contents, leading to a reduction in soil microorganism contents [57]. In contrast, high N inputs provided more nutrients for soil microorganisms, promoting their growth and reproduction [58]. The present study also found that low N deposition increased the content of G^+^ while high N deposition decreased it, which is in line with the previous findings that the increase in soil available N under low N deposition conditions may alleviate the N limitation of soil microorganisms and promote the metabolic activities of G^+^ fine to SOC, whereas excessive N inputs may disrupt the balance of the soil microbial community and threaten the soil health [59,60]. The ratio of soil F/B reflects soil fertility and health and can be used to characterize the responses of soil microorganisms to their surroundings and the ecosystem stability [16,61]. In the present study, we found that increased N deposition reduced the soil F/B in both years. The negative effects of increased N deposition on soil fungal communities may be due to the lower N requirement of soil fungi compared to soil bacteria, and thus, higher soil N effectiveness after increased N deposition would favor soil bacteria over soil fungi [13,62]. In addition, due to the harsh environmental conditions with soil water and N deficiencies in desert ecosystems, increased precipitation and N deposition can significantly promote soil microorganisms’ activities [16,63]. We found that the contents of soil fungi, DSE, Act, and bacteria were significantly increased under increased precipitation with increased N deposition in 2016, which is inconsistent with the existing studies. The previous studies indicated that increased N deposition promoted microorganisms’ nitrification reactions and induced soil acidification by directly increasing soil NO_3_^−^-N and NH_4_^+^-N concentration, increasing ionic toxicity in the soil, and in turn reducing the soil microbial community diversity and biomass [19,64]. However, it has also been proven that increased precipitation and N deposition have positive effects on the soil microbial community, which is consistent with our results [65]. The possible reason is that with increasing levels of precipitation and N deposition, the SOC content gradually increased, providing more organic carbon and N sources for soil microorganisms, thus promoting the growth and reproduction of soil microorganisms [66,67]. Furthermore, changes in soil G^+^/G^−^ indicate the soil nutrient status and soil substrate quality [68], In this study, we found that increased precipitation, increased N deposition, and their interactions increased the soil G^+^/G^−^ ratio, probably because soil G^−^ preferentially uses the fresh organic inputs as carbon sources, while soil G^+^ is thought to be favor of the low-quality or recalcitrant organic matter [69,70].

Increased N deposition has been widely recognized as one of the main factors contributing to the decline in ecosystem biodiversity, in which the diversity of soil microbial communities is also significantly affected [11,71]. In this study, it was found that soil microbial community diversity showed a tendency to decrease and then increase with increasing levels of N deposition, which is consistent with previous studies [19,72]. These results indicate that the effects of increased N deposition on soil microbial community diversity are threshold-limited, i.e., soil microbial community diversity changes from decreasing to increasing when N is added up to a certain threshold. While one recent study in the Inner Mongolian grasslands found that the Shannon diversity and Pielou evenness decreased under high N deposition, which is inconsistent with our results [73]. This may be due to the fact that high N treatments could induce soil acidification, altering soil physicochemical properties which affect soil aeration, water retention capacity, and nutrient adsorption capacity, and resulting in negative effects on soil microbial community structure [73,74]. Secondly, soil microorganisms could be difficult to maintain their reproductive needs due to the insufficient N supply, leading to decreases in soil microbial community diversity under low N deposition levels [75]. In contrast, with the increase in N deposition, the proportion of soil effective nutrients changed, and the competition for N between soil microorganisms and plants was weakened, allowing soil microorganisms to obtain sufficient N sources to support their growth and reproduction, leading to the enhancement of soil microbial community diversity under high N deposition conditions [76].

This study reveals the potential influence mechanisms of increased precipitation and N deposition on soil microbial communities, providing a scientific basis for the sustainable management of desert ecosystems [26]. Based on this, it is recommended to set a risk-graded threshold of N deposition for the ecological management in desert ecosystems. And we should further prioritize the control of anthropogenic N inputs through the promotion of slow-release N fertilizers when regional deposition is lower than this threshold. When the threshold is exceeded, it is necessary to mitigate soil acidification in combination with the application of biochar or lime in order to maintain the balance of microbial functions [11]. Thus, the synergistic promotion of N emission reduction and carbon neutrality will be realized. In addition, plants also pose significant impacts on soil microorganisms, mainly through their ability to cause changes in soil physicochemical properties, combining the effects of plant community diversity, which indirectly influence soil microbial community structure by altering the micro-environment in which soil microorganisms grow [52,53]. However, our current study did not monitor the dynamic changes in plant diversity and cover, future studies need to integrate multi-scale observations. This could be achieved by combining hyperspectral remote sensing to invert vegetation cover and the NDVI index, quantifying three-dimensional structural features of plant communities through unmanned aerial photography, to reveal plant-mediated indirect effects on soil microbial communities. Based on this, further research should focus on exploring the direct effects of global climate change on soil microbial communities by influencing soil properties, and, at the same time, revealing the indirect effects of combining with changes in plant communities to comprehensively reveal the effects of global climate change on soil microbial communities [27].

## 5. Conclusions

In this study, the underlying influence mechanisms of the effects of increased precipitation and N deposition on the soil microbial community structure were deeply analyzed using the PLFA technique in desert ecosystems. We found that increased precipitation and N deposition showed different effects on soil microorganism groups in different natural precipitation years. In a normal precipitation year, increased precipitation decreased soil fungi and Act content, but increased soil bacterial content. In contrast, in a drought year, increased precipitation promoted the growth of soil fungi and Act content. Moreover, the interactions of increased precipitation and N deposition posed negative effects on the soil Act content. Increased N deposition significantly decreased the soil F/B ratio, and increased precipitation interacted with N deposition to increase the soil G^+^/G^−^ ratio in both years. Neither increased levels of precipitation nor increased N deposition had a significant effect on soil microbial community diversity in both years. Based on the random forest and linear regression analyses, soil pH and MBC were the main factors affecting soil microbial community structure. Finally, SEM was employed to further elucidate the mechanisms of the effects of increased precipitation and N deposition on the soil microbial community. The best-fitting SEM showed that increased precipitation increased soil G^+^ content, and decreased soil Act, fungi, and DSE content by decreasing soil MBC; increased N deposition decreased soil AMF content, but increased soil DSE content by decreasing soil pH in the normal precipitation year. In the drought year, increased precipitation decreased soil G^+^, G^−^, Act, fungi, AMF, and DSE content by decreasing soil MBC. In summary, increased precipitation and N deposition showed significant synergistic effects on soil microbial community structure by altering soil pH and MBC, and the effects differed under different natural precipitation conditions in desert ecosystems. In addition, future studies could incorporate macro-genomics to reveal metabolic pathway adaptations in key functional taxa and quantify the impacts of soil microbial communities through stable isotopes to assess the long-term response of desert ecosystem carbon sink functions to global change.

## Figures and Tables

**Table 1:** Effects of increased precipitation and N deposition on soil physicochemical properties in 2016 and 2017, respectively. All data are presented as the mean ± standard error. The ANOVA results are shown in Table S1.

Year	Treatment	pH	SOC	NO_3_^−^-N	NH_4_+-N	MBC	MBN
			(g/kg)	(mg/kg)	(mg/kg)	(mg/kg)	(mg/kg)
2016	W0N0	8.75 ± 0.12 a	2.14 ± 0.08 bc	4.40 ± 0.31 d	1.57 ± 0.35 c	168.33 ± 30.14 bc	66.43 ± 3.90 bc
	W0N1	8.55 ± 0.02 ab	2.37 ± 0.09 b	6.19 ± 0.12 bc	6.99 ± 1.79 ab	181.37 ± 18.10 ab	68.54 ± 2.37 b
	W0N2	8.47 ± 0.06 b	2.70 ± 0.08 a	8.91 ± 0.28 a	8.66 ± 1.47 a	223.87 ± 6.20 a	76.62 ± 1.56 a
	W1N0	8.73 ± 0.10 ab	2.06 ± 0.03 c	6.37 ± 0.29 b	3.01 ± 0.55 c	160.03 ± 11.84 bc	65.72 ± 1.64 bc
	W1N1	8.65 ± 0.11 ab	2.19 ± 0.04 bc	6.89 ± 0.29 b	4.04 ± 0.74 bc	164.77 ± 7.51 bc	66.08 ± 1.56 bc
	W1N2	8.49 ± 0.04 ab	2.76 ± 0.13 a	5.54 ± 0.23 c	9.94 ± 1.04 a	121.55 ± 19.20 c	60.17 ± 1.45 c
2017	W0N0	8.70 ± 0.11 a	2.09 ± 0.06 bc	4.29 ± 0.21 d	1.52 ± 0.42 c	161.20 ± 31.57 bc	63.10 ± 3.08 bc
	W0N1	8.57 ± 0.02 a	2.41 ± 0.10 ab	6.04 ± 0.16 bc	6.93 ± 1.71 ab	183.26 ± 21.83a b	67.11 ± 3.15 b
	W0N2	8.50 ± 0.11 a	2.56 ± 0.17 a	8.79 ± 0.45 a	8.20 ± 1.75 a	227.28 ± 7.37 a	74.43 ± 1.42 a
	W1N0	8.53 ± 0.11 a	2.03 ± 0.05 c	6.14 ± 0.26 bc	2.76 ± 0.68 c	163.97 ± 11.20 bc	64.26 ± 1.61 bc
	W1N1	8.75 ± 0.17 a	2.14 ± 0.04 bc	6.61 ± 0.34 b	3.68 ± 0.90 bc	162.88 ± 8.63 bc	64.46 ± 1.44 bc
	W1N2	8.50 ± 0.01 a	2.57 ± 0.15 a	5.46 ± 0.29 c	8.96 ± 1.64 a	119.28 ± 16.97 c	59.65 ± 2.03 c

Note: Different lowercase letters in the same column indicate significant differences between treatments (*p* < 0.05). SOC, Soil organic carbon; NO_3_^−^-N, Nitrate nitrogen; NH_4_^+^-N, Ammonium nitrogen; MBC, Microbial biomass carbon; MBN, Microbial biomass nitrogen; W0, Natural precipitation; W1, 30% increase in natural precipitation; N0, Natural N deposition; N1, Medium N deposition; N2, High N deposition.

## Data Availability

The original contributions presented in this study are included in the article/Supplementary Materials, further inquiries can be directed to the corresponding author.

## Supplementary Materials

The following supporting information can be downloaded at: https://www.mdpi.com/article/10.3390/microorganisms13061393/s1, Figure S1: Location map of the study area; Figure S2: Changes in total precipitation and average temperature from 2016 to 2017; Figure S3: Effects of increased precipitation and N deposition on the relative abundance of each soil microbial groups in 2016 and 2017, respectively; Figure S4: Effects of increased precipitation and N deposition on soil microbial community in 2016 and 2017 based on non-metric multidimensional scaling (NMDS) analysis; Figure S5: Relationships between soil pH and each group of soil microorganisms PLFA content soil microorganisms in 2016 and 2017, respectively; Figure S6: Relationships between soil MBC and each group of soil microorganisms PLFA content soil microorganisms in 2016 and 2017, respectively; Figure S7: Structural equation modeling (SEM) illustrates the causal pathways by which increased precipitation and increased N deposition affected the soil microbial community in 2016 and 2017, respectively; Table S1: Results of two-way factorial ANOVA on the effects of increased precipitation (W) and nitrogen deposition (N) on soil physicochemical properties in 2016 and 2017, respectively; Table S2: Results of two-factor ANOVA for Soil microbial PLFA content under increased precipitation and increased N deposition, 2016 and 2017; Table S3: Results of two-factor ANOVA for Soil microbial PLFA content under increased precipitation and increased N deposition, 2016 and 2017; Table S4: Results of two-factor ANOVA analysis of soil microbial diversity under increased precipitation and increased N deposition, 2016 and 2017; Table S5: Results of changes in soil microbial diversity under increased precipitation and N deposition in 2016 and 2017.
