# Supplementary material for: Interactive Effects of Precipitation and Nitrogen on Soil Microbial Communities in a Desert Ecosystem
Source: Microorganisms. 2025 Jun 14;13(6):1393. doi: 10.3390/microorganisms13061393 (PMC12196170; doi:10.3390/microorganisms13061393)
Supplement: Supplementary file 1 [file microorganisms-13-01393-s001.zip › microorganisms-3692566-supplementary.pdf]

microorganisms

MDPI

Supplementary Materials

Table S1. Results of two-way factorial ANOVA on the effects of increased precipitation (W) and nitrogen deposition (N) on soil physicochemical properties in 2016 and 2017, respectively. *p* values in bold are significantly different ($p < 0.05$).

year	treatment	PH	SOC (g/kg)	NO_3^--N (mg/kg)	NH_4^+-N (mg/kg)	MBC (mg/kg)	MBN (mg/kg)
2016	W	0.65	0.32	0.30	0.936	***p*<0.05**	***p*<0.05**
	N	***p*<0.05**	***p*<0.05**	***p*<0.05**	***p*<0.05**	0.85	0.60
	W*N	0.74	0.36	***p*<0.05**	0.11	***p*<0.05**	***p*<0.05**
2017	W	0.99	0.26	0.24	0.70	***p*<0.05**	***p*<0.05**
	N	0.33	***p*<0.05**	***p*<0.05**	***p*<0.05**	0.80	0.34
	W*N	0.26	0.44	***p*<0.05**	0.20	***p*<0.05**	***p*<0.05**

Note: SOC, Soil organic carbon; NO_3^--N, Nitrate nitrogen; NH_4^+-N, Ammonium nitrogen; MBC, Microbial biomass carbon; MBN, Microbial biomass nitrogen. W, Increased precipitation; N, Nitrogen deposition; W*N, Increased precipitation and nitrogen deposition interaction.

Table S2. Results of two-factor ANOVA for Soil microbial PLFA content under increased precipitation and increased N deposition, 2016 and 2017. *p* values in bold are significantly different ($p < 0.05$).

year	treatments	G^+	G^-	G^+/G^-	Fungi	AMF	DSE	Actinomycetes	Bacteria	F/B	Total
2016	W	0.31	0.82	0.93	0.17	0.13	0.26	0.08	0.52	0.13	0.27
	N	0.17	0.69	0.35	0.90	0.31	0.42	0.38	0.51	0.7	0.90
	W*N	0.07	0.46	0.46	0.08	0.35	0.32	0.32	0.4	0.17	0.34
2017	W	0.67	0.65	0.49	0.70	0.46	0.80	0.27	0.96	0.78	0.91
	N	0.59	0.11	0.77	0.10	0.60	0.32	0.46	0.24	0.43	0.19
	W*N	0.69	0.27	0.17	0.99	0.37	0.87	0.05	0.82	0.70	0.82

Note: G^+, Gram-positive bacteria; G^-, Gram-negative bacteria; G^+/G^-, Gram-positive bacteria/Gram-negative bacteria; AMF, Arbuscular Mycorrhizal Fungi; DSE, Dark Septate Endophyte; F/B, Fungi/Bacteria.

Table S3. The results of changes in soil microbial contents under increased precipitation and N deposition in 2016 and 2017, respectively. All data are presented as the mean ± standard error. (nmol/g).

Year	Treatment	Gram-positive bacteria	Gram-negative bacteria	G^+/G^-	Fungi	AMF	DSE	Actinomycetes	Bacteria	F/B	Total
2016	W0N0	0.73±0.07ab	0.48±0.13a	1.78±0.37a	0.68±0.11a	0.20±0.10a	0.54±0.08a	0.34±0.05a	1.21±0.17a	0.65±0.21a	2.96±0.10a
	W0N1	0.76±0.06a	0.66±0.11a	1.21±0.15a	0.53±0.08a	0.07±0.01a	0.38±0.05a	0.17±0.07b	1.42±0.14a	0.37±0.03a	2.56±0.33a
	W0N2	0.56±0.06b	0.60±0.07a	0.96±0.17a	0.71±0.11a	0.12±0.03a	0.56±0.10a	0.23±0.05ab	1.16±0.09a	0.64±0.14a	2.78±0.20a
	W1N0	0.63±0.06ab	0.61±0.08a	1.06±0.07a	0.47±0.07a	0.08±0.02a	0.38±0.07a	0.15±0.03b	1.24±0.13a	0.38±0.02a	2.31±0.30a
	W1N1	0.80±0.03a	0.54±0.10a	1.72±0.47a	0.63±0.10a	0.08±0.01a	0.44±0.08a	0.17±0.03b	1.34±0.10a	0.48±0.10a	2.65±0.17a
	W1N2	0.77±0.08a	0.66±0.10a	1.23±0.16a	0.52±0.05a	0.07±0.01a	0.46±0.05a	0.18±0.07ab	1.43±0.15a	0.37±0.03a	2.66±0.32a
2017	W0N0	0.24±0.03a	0.30±0.03ab	0.78±0.05a	0.27±0.01a	0.04±0.01a	0.26±0.02a	0.06±0.01ab	0.54±0.06a	0.51±0.04a	1.17±0.09a
	W0N1	0.19±0.02a	0.22±0.02b	0.88±0.05a	0.20±0.03a	0.03±0.01a	0.20±0.03a	0.05±0.01b	0.42±0.04a	0.50±0.07a	0.89±0.09a
	W0N2	0.21±0.04a	0.31±0.03ab	0.66±0.09a	0.24±0.03a	0.04±0.01a	0.25±0.03a	0.10±0.01a	0.53±0.07a	0.46±0.03a	1.16±0.13a
	W1N0	0.19±0.05a	0.32±0.02a	0.61±0.17a	0.28±0.04a	0.03±0.01a	0.25±0.06a	0.10±0.02ab	0.51±0.05a	0.56±0.06a	1.16±0.16a
	W1N1	0.18±0.05a	0.28±0.04ab	0.67±0.18a	0.21±0.04a	0.03±0.01a	0.21±0.04a	0.08±0.02ab	0.45±0.08a	0.46±0.05a	0.99±0.17a
	W1N2	0.23±0.02a	0.27±0.02ab	0.85±0.02a	0.24±0.02a	0.04±0.01a	0.24±0.02a	0.07±0.01ab	0.51±0.04a	0.48±0.03a	1.10±0.07a

Note: Different lowercase letters in the figure indicate significant differences between treatments ($p < 0.05$). W0, Natural precipitation; W1, 30% increase in natural precipitation; N0, Natural deposition; N1, Medium N deposition; N2, High N deposition.

Table S4. Results of two-factor ANOVA analysis of soil microbial diversity under increased precipitation and increased N deposition, 2016 and 2017. *p* values in bold are significantly different ($p < 0.05$).

year	treatment	Shannon-Wiener index	Simpson index	Pielou index
2016	W	0.12	0.15	0.12
	N	0.06	**$p<0.05$**	0.06
	W*N	0.32	0.42	0.32
2017	W	0.32	0.26	0.32
	N	0.34	0.40	0.34
	W*N	0.87	0.83	0.87

Table S5 Results of changes in soil microbial diversity under increased precipitation and N deposition in 2016 and 2017.

year	treatment	**Shannon-Wiener index**	**Simpson index**	**Pielou index**
2016	W0N0	1.66±0.03a	0.80±0.01a	0.93±0.02a
	W0N1	1.56±0.03b	0.77±0.01b	0.87±0.02b
	W0N2	1.64±0.02ab	0.79±0.01ab	0.91±0.01ab
	W1N0	1.61±0.02ab	0.78±0.01ab	0.90±0.01ab
	W1N1	1.57±0.02b	0.77±0.01b	0.87±0.01b
	W1N2	1.58±0.03b	0.77±0.01ab	0.88±0.02b
2017	W0N0	1.61±0.01a	0.78±0.01a	0.90±0.01a
	W0N1	1.61±0.01a	0.78±0.02a	0.90±0.01a
	W0N2	1.64±0.01a	0.79±0.03a	0.92±0.01a
	W1N0	1.57±0.05a	0.77±0.02a	0.88±0.02a
	W1N1	1.60±0.04a	0.77±0.02a	0.89±0.02a
	W1N2	1.62±0.01a	0.79±0.01a	0.91±0.01a

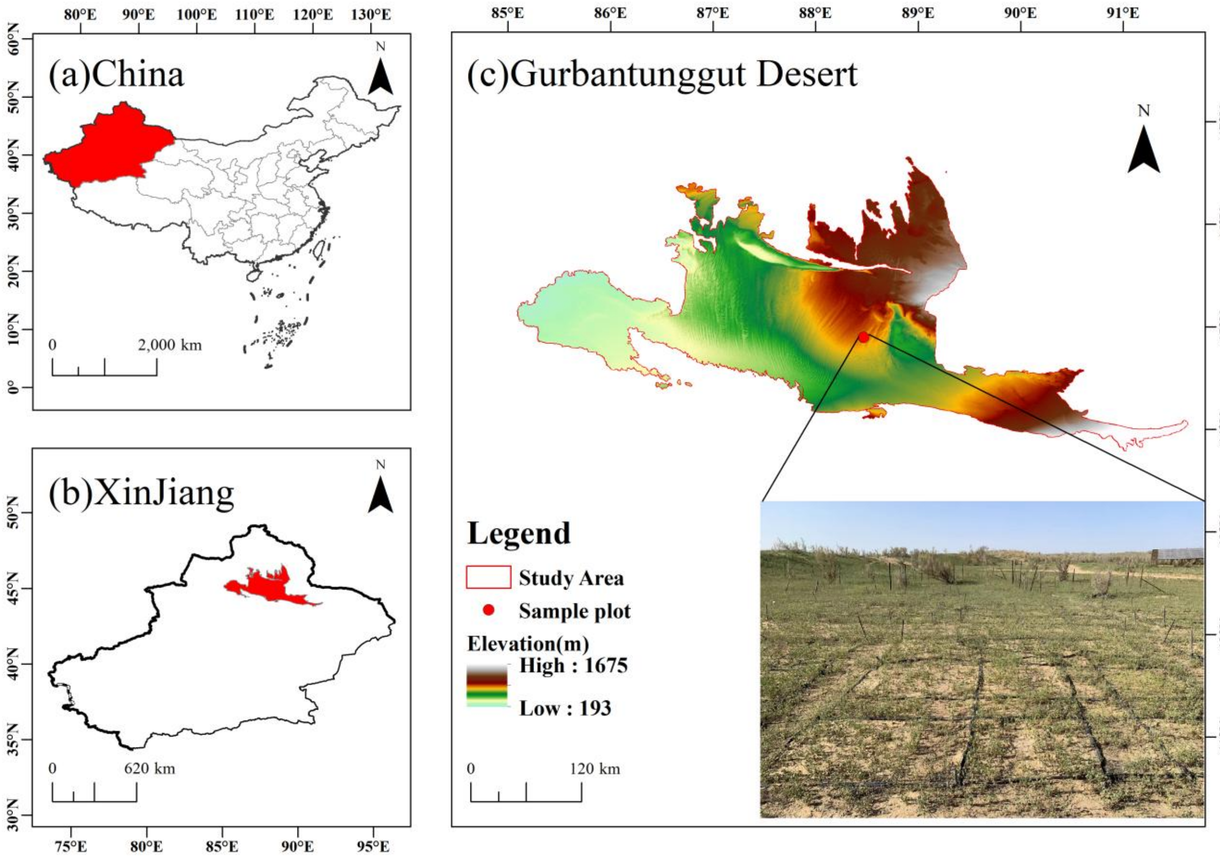

Figure S1 Location map of the study area: geographic location of Xinjiang (a); geographic location of the Gurbantunggut Desert (b); geographic location of sampling sites in the study area (c).

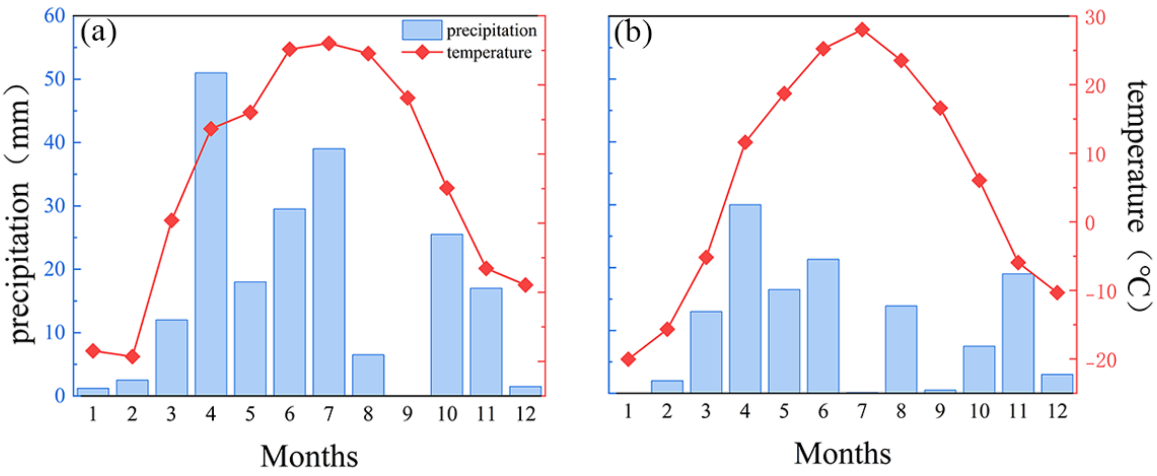

Figure S2 Changes in total precipitation and average temperature from 2016 (a) to 2017 (b). Bar charts and line graphs represent the trends in precipitation and temperature, respectively.

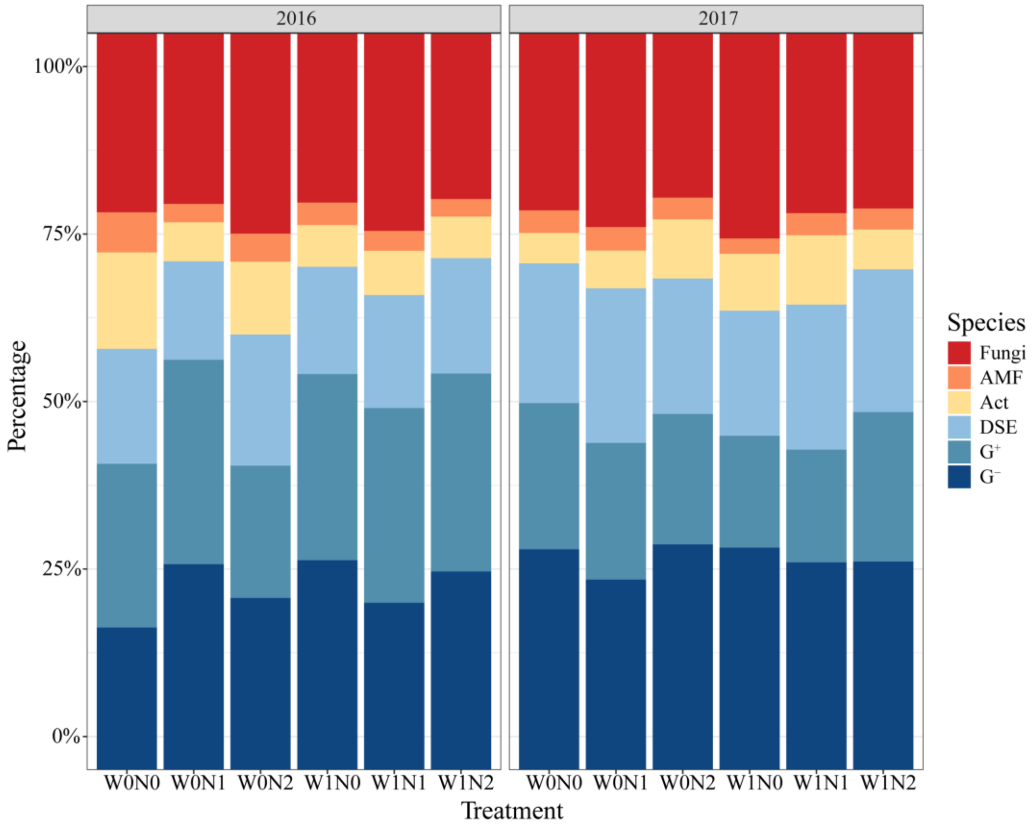

Figure S3. Effects of increased precipitation and N deposition on the relative abundance of each soil microbial groups in 2016 and 2017, respectively. Mean values are shown on the figure. Note: W0, Natural precipitation; W1, 30% increase in natural precipitation; N0, Natural sedimentation; N1, Medium N deposition; N2, High N deposition. SOC, Soil organic carbon; NO_3^--N, Nitrate nitrogen; NH_4^+-N, Ammonium nitrogen; MBC, Microbial biomass carbon; MBN, Microbial biomass nitrogen.

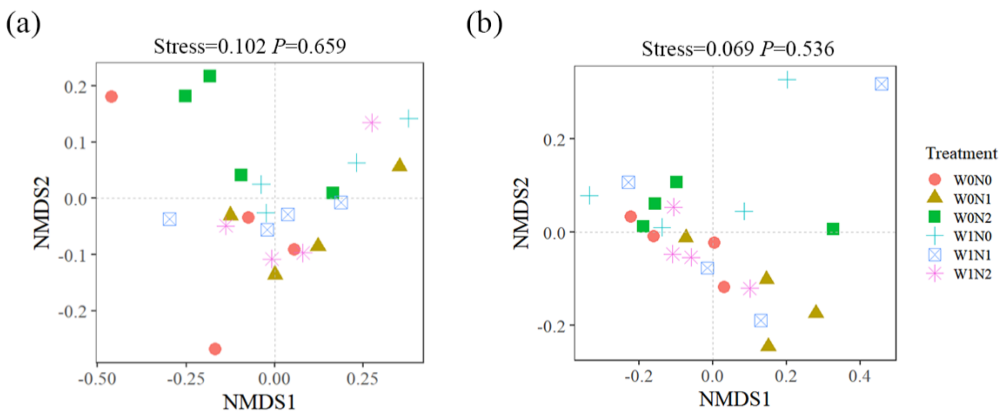

Figure S4. (a,b) Effects of increased precipitation and N deposition on soil microbial community in 2016 and 2017 based on non-metric multidimensional scaling (NMDS) analysis.

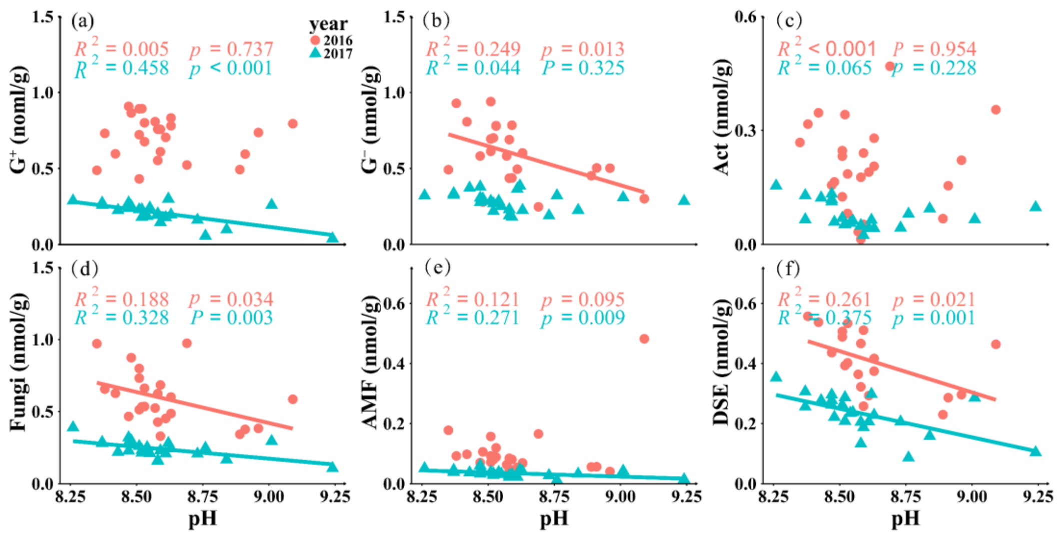

Figure S5. (a-f) Relationships between soil pH and each group of soil microorganisms PLFA content soil microorganisms in 2016 (red) and 2017 (blue), respectively. The solid line indicated the signifi-cant relationships with $p < 0.05$.

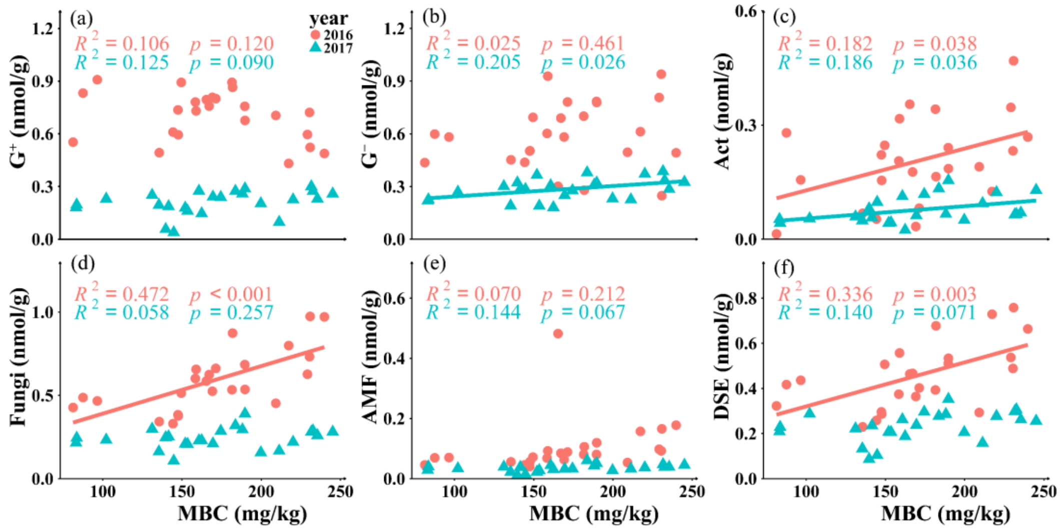

Figure S6. (a-f) Relationships between soil MBC and each group of soil microorganisms PLFA content soil microorganisms in 2016 (red) and 2017 (blue), respectively. The solid line indicated the signifi-cant relationships with $p < 0.05$.

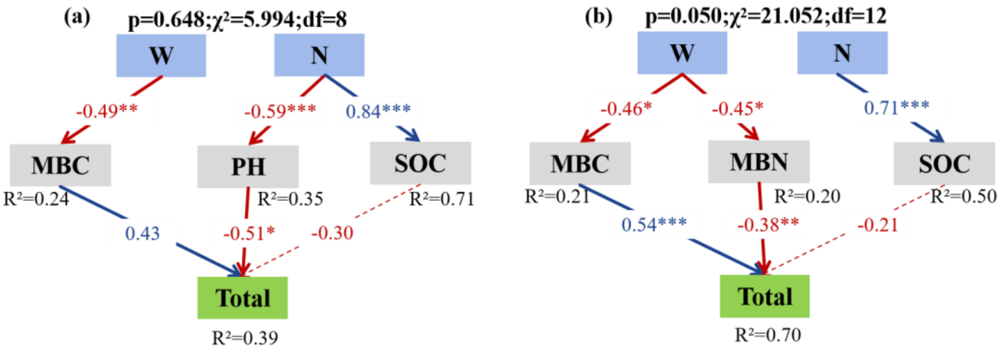

Figure S7. Structural equation modeling (SEM) illustrates the causal pathways by which increased precipitation and increased N deposition affected the soil microbial community in 2016 (a) and 2017 (b), respectively. Solid red and blue arrows indicate negative and positive significant effects, respectively, and dashed lines indicate non-significant effects. Numbers above the arrows indicate the magnitude of the standardized SEM coefficients (*, $p < 0.05$; **, $p < 0.01$; ***, $p < 0.001$). R^2 values indicate the proportion of variance explained by each variable.
